# Chaperone-dependent Neurodegeneration: A Molecular Perspective on Therapeutic Intervention

**DOI:** 10.4172/2161-0460.S10-007

**Published:** 2013-04

**Authors:** Aaron Carman, Sarah Kishinevsky, John Koren, Wenjie Lou, Gabriela Chiosis

**Affiliations:** 1 Department of Molecular Pharmacology and Chemistry, Memorial Sloan-Kettering Cancer Centre, New York, NY, USA; 2 Department of Neurology and Neuroscience, Weill Cornell Medical College, New York, NY, USA

**Keywords:** Neurodegeneration, Molecular chaperones, Aberrant neurological proteins, Chaperone inhibitors

## Abstract

Maintenance of cellular homeostasis is regulated by the molecular chaperones. Under pathogenic conditions, aberrant proteins are triaged by the chaperone network. These aberrant proteins, known as “clients,” have major roles in the pathogenesis of numerous neurological disorders, including tau in Alzheimer’s disease, α-synuclein and LRRK2 in Parkinson’s disease, SOD-1, TDP-43 and FUS in amyotrophic lateral sclerosis, and polyQ-expanded proteins such as huntingtin in Huntington’s disease. Recent work has demonstrated that the use of chemical compounds which inhibit the activity of molecular chaperones subsequently alter the fate of aberrant clients. Inhibition of Hsp90 and Hsc70, two major molecular chaperones, has led to a greater understanding of how chaperone triage decisions are made and how perturbing the chaperone system can promote clearance of these pathogenic clients. Described here are major pathways and components of several prominent neurological disorders. Also discussed is how treatment with chaperone inhibitors, predominately Hsp90 inhibitors which are selective for a diseased state, can relieve the burden of aberrant client signaling in these neurological disorders.

## Introduction

### Chaperones in disease

**Figure 1:**
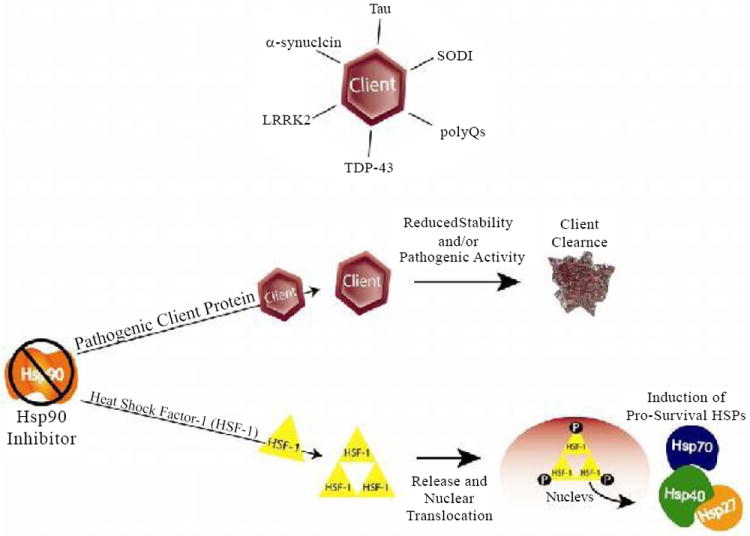
Inhibition of Hsp90 generates two distinct mechanisms both of which result in client protein degradation and enhanced cell survival. Pathogenic Hsp90 “client” proteins are dependent on Hsp90 for nascent folding and maintenance of structure. Upon Hsp90 inhibition, client proteins, including those involved in disease lose stability and are degraded. Hsp90 inhibition also promotes the induction of other heat shock proteins through an HSF1 dependent mechanism. These HSPs can also promote clearance and block aggregation of aberrant clients while enhancing cell survival under the “stressed” condition.

Eukaryotes have evolved elaborate systems of chaperone proteins to cope with cellular stress. Under normal cellular conditions, chaperones regulate nascent protein folding. Cellular stressors, such as a thermal stress, can cause proteins to become unfolded, non-functional, or even structurally damaged. To maintain cellular homeostasis, the chaperone network will interact with these damaged “client” proteins and attempt to fold the protein back into a functional state or target the client for degradation. However, certain mutated or abnormally-modified disease-causing proteins are stabilized and maintained by the multitasking heat-shock protein 90 (Hsp90) and its co-chaperone network [1-3]. Such is the case in certain cancers, where the Hsp90 chaperone network facilitates disease by stabilizing oncogenic client proteins. It is now becoming clear that Hsp90 and its co-chaperones also regulate a majority of neurodegenerative proteinopathy (Figure 1 and Table 1).

A role for Hsp90 in the maintenance of neurodegenerative diseases is thought to be similar to its proposed role in cancer: mis-folding or stabilization of aberrant (neurotoxic) client- proteins. Because Hsp90 client-protein folding (and stabilization) is ATP-dependent, Hsp90 activity can be manipulated by ATP competitive small molecules. Indeed, the first known Hsp90 inhibitor, the antibiotic geldanamycin (GA), was discovered to compete with ATP for binding which stops the protein folding cycle and prevents client stabilization [4-7].

While these important biological roles propose a role for Hsp90 inhibitors in the treatment of neurodegenerative disease, it is also true that Hsp90 is one of the most abundantly (~1-3% of total cellular protein) and ubiquitously expressed proteins. Such finding matches poorly with the belief that a good therapeutic target has to be of low expression in vital organs and tissues. Using the GA derivative 17-allylaminogeldanamycin (17-AAG), Kamal et al. provided the rationale for a therapeutic index for the use of Hsp90 inhibitors in disease. Specifically, they demonstrated the presence of high-ATP-affinity Hsp90 complexes seemingly exclusive to diseased cells. High-affinity species bound to these small-molecule ATPase inhibitors with nearly 100-fold higher affinity than Hsp90 from non-cancer cells [8]. This finding made GA, and similar inhibitors, both potently selective and therapeutically attractive.

Further work by Moulick et al. demonstrated that only a small percentage of the total cellular Hsp90 population exists in this high-affinity state [9]. It is thought that as the levels of intracellular aberrant proteins rise, so does the need for Hsp90; the cell’s attempt to deal with accumulating proteins depends on greater Hsp90 activity. While the complete nature and composition of this high-affinity Hsp90 complex remain unclear, post-translational modifications [10] and binding of co-chaperones [9] to Hsp90 are likely involved. The shift to greater ATP- affinity and increased activity promotes the stabilization of pools of aberrant protein clients, possibly by passing them through folding cycles that are ultimately unproductive, stabilizing species whose fate should be degradation. This process facilitates and maintains many cancers and, as we show further here, many neurodegenerative diseases as well.

Aberrant proteins become clients of Hsp90 or are regulated by the Hsp90 chaperone network [9]. As mutant proteins continue to be experimentally recapitulated in cellular and animal models, much has been learned about the pivotal role of Hsp90 and its extensive co- chaperone network in the stabilization of aberrant client proteins. We will begin this review by examining tau as a model Hsp90 client, including the crucial role of Hsp70 in tau pathology.

**Figure 2:**
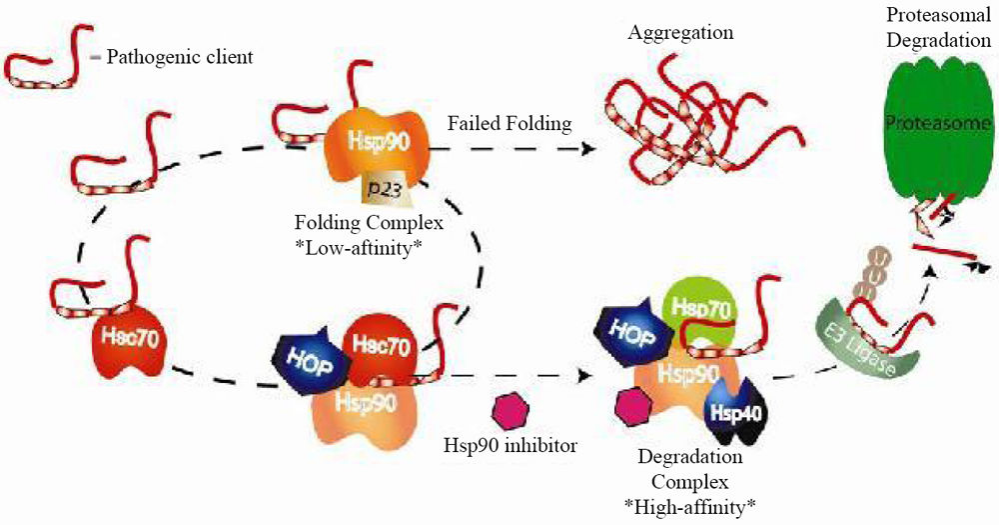
Protein folding pathways can prove to be detrimental when the chaperone folding machinery fails to properly process an aberrant aggregation-prone client protein. Under the condition of Hsp90 inhibition, Hsp90 is removed from the unproductive folding equation, disrupting this pro-aggregation pathway. Also, inhibition of Hsp90 induces the HSPs, including Hsp70. Increased levels of Hsp70 can facilitate a higher incident of interaction between client and pro-degradation pathway. As an example, interaction of a client with an ubiquitin ligase promotes proteasomal degradation of aberrant clients rather than accumulation and aggregation.

These ideas will serve as a framework for subsequent discussion of aberrant proteins in other neurodegenerative diseases. We will evaluate experimental results from various cellular and animal disease models and discuss the therapeutic potential of pharmacologic intervention at the chaperone level. Lastly, we will propose a general model of chaperone dependency that links much seemingly-disparate neurodegenerative pathologies (Figure 2).

## Hsp90/Chaperone-dependent Tauopathies

### Tau in neurodegeneration

The microtubule associated protein tau (MAPT) is a focal point of cellular regulation that integrates and responds to input from converging signaling cascades to regulate microtubule dynamics. Loss of microtubule function is detrimental to multiple neuronal systems including axonal transport, mitochondrial function, and autophagy [11-13]. In disorders featuring aberrant tau, a group collectively known as tauopathies, microtubule dysfunction is a common feature and this dysfunction has been suggested to be an initiating factor for the accumulation of tau. Tau affinity for microtubules is largely regulated by site-specific phosphorylation. The N-terminal acidic domain and C-terminal microtubule binding domain of tau flank a proline-rich middle region that is a target for pathogenic hyperphosphorylation by many proline-directed tau kinases. Tau exists as six isoforms each of which has a different affinity for microtubules [14,15]. Two predominant groups are comprised of tau isoforms containing 3 or 4 microtubule-binding domain repeats in the C-terminus (3R or 4R). Tau mutations altering the 3R/4R tau ratio, normally about 1:1 [16], perturb tau proteostasis and can lead to microtubule destabilization and disease [17].

The phosphorylation and isoform specifics of tau are not the only factors that may drive tau pathogenicity. Tau mutations are responsible for a diverse group of neurodegenerative dementias that include Pick’s disease, progressive supranuclear palsy (PSP) [18,19], corticobasal degeneration (CBD) [20-22], and fronto-temporal dementia with parkinsonism linked to chromosome-17 (FTDP-17), a rare but devastating disease [23]. These disease-linked tau mutations affect tau phosphorylation and dephosphorylation [24,25], which then alter tau affinity for microtubules [26-32]. These mutations also change the predilection of tau to aggregate into paired-helical filaments (PHF) and neurofibrillary tangles (NFT) [33-35]. For example tauP301L and tauP301S, two commonly-studied mutations, have each been linked to FTDP-17 [23,36,37]. Transgenic mice expressing human tau with these mutations accumulate hyperphosphorylated, insoluble tau and undergo cognitive and motor decline [38-44]. To date, more than 37 tau mutations have been associated with FTDP-17 [45]. Although NFTs comprised of insoluble tau are a hallmark of Alzheimer’s disease, no tau mutations have yet been associated with AD. Finally, accumulation of tau and other neuronal disease-related proteins is thought to be exacerbated by age-dependent decline in protein degradation, as deficits in proteasomal processing and autophagy are observed in aging brains [46].

Perhaps the most remarkable aspect to tau pathogenesis is the fact that mutant tau fibrils can spread from cell to cell and confer a pathogenic conformation to normal tau, a process similar to intercellular prion seeding [47,48]. Upon selective expression of tauP301L in the entorhinal cortex of mice, the origin of tau pathology in AD [49], pathogenic tau spread trans- synaptically through neuronal circuits in a temporal and spatial progression that mimics human disease [50]. These findings support the importance of developing reliable early diagnostic techniques for tau and also point to multiple steps of potential therapeutic intervention. As we shall see, they also raise questions about which steps are facilitated by chaperones.

In neurons, the breadth of tau function underlies the impact of its loss, or the loss of its normal functions. Once thought to be without phenotype because of redundancy found in related microtubule-binding proteins, tau knockout mice were recently shown to develop age- dependent brain atrophy and “parkinsonism-like” symptoms [51]. Interestingly, this degeneration was associated with a diminished neuronal iron transport mediated by amyloid precursor protein (APP), the protein processed into the aggregation-prone β-amyloid (Aβ) in Alzheimer’s disease. Although neither APP nor Aβ has been confirmed as a bone fide Hsp90 client, *in vitro* studies suggest Aβ peptide aggregation can be influenced by Hsp90 and its co-chaperone Hsp70 [52,53]. Additionally, studies in HEK cells suggest CHIP, Hsp70 and Hsp90 all participate in APP metabolism [54]. The Hsp90 chaperone network might also regulate drivers of Aβ production or aggregation [54,55]. Importantly, most current models of AD pathology support a role for Aβ as a driver of tau pathology [56,57] and a role for tau as the primary mediator of accumulating Aβ toxicity [58-60]. While the exact role of Hsp90 in Aβ pathology in AD remains unclear, Hsp90 and its co-chaperones play critical roles in facilitating tau pathology.

### Hsp70/90 regulation of Tau proteostasis

The first clues to Hsp70 and Hsp90 involvement in tau/microtubule regulation came in 2003 with the demonstration that higher levels of Hsp70 and Hsp90 correlated with lower levels of insoluble tau and increased tau-microtubule association [61]. In 2007, “high-ATP-affinity” Hsp90 complexes were discovered in the temporal cortex (an affected area) but not in the cerebellum (an unaffected area) of post-mortem brains from AD patients [62].

Before tau encounters Hsp90, though, it is bound by either Hsc70 or Hsp70, which control tau access to the proteasomal degradation machinery, the “triage decision”. Following microtubule destabilization, tau first binds to the constitutively-expressed co-chaperone heat-shock cognate protein-70, Hsc70 [63]. Once this complex has formed, the aberrant client protein faces one of two fates: either folding or degradation. The client could be presented to Hsp90 through the scaffolding co-chaperone HOP (Hsp70/Hsp90 organizing protein) for attempted folding. Hsp90 interaction with either Hsc70 or Hsp70 may play opposing roles in facilitating accumulation of problem clients like tau: Hsc70 binding appears to stabilize tau and shelter it from degradation [63]. Also, the multi-functional modulator Bcl2-associated athanogene-1 (BAG-1) associates with the tau-Hsc70 complex and actually prevents proteasomal degradation of tau, a situation reversed by Hsp70 induction [64]. Hsp70-bound tau can be ubiquitinated by the E3-ubiquitin ligase CHIP (Hsc70-interacting protein) and targeted for proteasomal degradation [62,65-68]. These finding suggest Hsp70 binding may favour tau degradation, whereas Hsc70 binding favours folding. Hsp70 and Hsc70 have been shown to have these opposing effects on other client proteins, as well [69]. These ideas are consistent with findings that Hsp70 differentially regulates tau isoforms and may function primarily in regulating dysfunctional tau isoforms [70].

Elegant biochemical studies by Kundrat and Regan demonstrate that CHIP binding to the Hsp70-client complex excludes HOP-Hsp90 binding [68]. Their in situ and *in vitro* analyses indicate that inhibition of Hsp90 causes client proteins to be degraded due to the expansion of the degradation pathway. Low cellular levels of Hsp70 and CHIP may control the basal levels of “house-keeping” turnover and degradation of tau, a pathway that is better accessed by tau when more Hsp70 is available to present tau to CHIP.

The potential for tau pathogenesis is also regulated by phosphorylation. This phospho- regulation of tau runs directly through the Hsp90 chaperone network. By chaperoning client kinases like glycogen synthase kinase (GSK3β), p35/CDK5 and microtubule affinity regulating kinase-2 (MARK2), three established tau kinases, the Hsp90 network controls the flow of signaling input to tau [53,71-73]. Hsp90 co-chaperones like the immunophilin FKBP51/2 regulate tau folding and mediate kinase access to tau [74,75], while another Hsp90 co- chaperone, protein phosphatase 5 (PP5), is a major tau phosphatase [73]. Thus Hsp90 acts as a nucleus for the complex regulatory machine that controls much of tau biology.

## Chaperone Inhibition in Alzheimer’s Disease and Tauopathy

### Inhibition of Hsp90

The most promising Hsp90 inhibitors target the N-terminal ATPase domain [76-79]. Many novel and synthetic Hsp90 inhibitors are already in clinical trials, but at this point they are being tested primarily in cancer [80-82]. Research in the last decade revealed the therapeutic potential of Hsp90 inhibition in proteinopathic neurodegeneration [83,84]. In early 2007, two studies demonstrated that Hsp90 inhibition decreases levels of hyperphosphorylated and/or mutated tau in cells and transgenic mice. Using the inhibitor EC102, Dickey et al. demonstrated selective degradation of hyperphosphorylated tau in both cells over-expressing tauP301L and in the human tau (hTau) mouse model [62,85], which expresses all 6 human isoforms of tau and develops NFT and “Alzheimer-like” pathology [86]. Additionally, Hsp90 inhibition with EC102 selectively decreased tau phosphorylated at Ser/Thr sites thought to be controlled by the proline-directed kinases, likely GSK-3β and Cdk5 [62,85,87,88]. Both kinases are known to participate in pathogenic tau phosphorylation while Akt, working with CHIP, also regulates tau degradation [89].

Also in early 2007, Luo et al. demonstrated that 17-AAG and the brain-permeable synthetic purine scaffold Hsp90 inhibitor, PU-DZ8, decreased levels of phosphorylated tau [90]. Importantly, not only does Hsp90 inhibition decrease levels of tau specifically phosphorylated on Ser202, a pathogenically-important site known to be phosphorylated by Cdk5, but also levels of the Cdk5-activator protein p35, which is itself a client of the Hsp90 chaperone network. Interestingly, while treatment of tauP301L mice with PU-DZ8 resulted in decreased soluble and insoluble tau, including mutant tau, treatment of hTau mice decreased Ser202-phosphorylated tau without affecting levels of endogenous tau [90].

As discussed, inhibition of Hsp90 in a system flooded with mis-folded client proteins has multiple beneficial effects. First, Hsp90 inhibition eliminates the folding pathway as an option for aberrant tau increasing the probability that tau will be ubiquitinated by CHIP and degraded. Second, Hsp90 inhibition initiates the HSF1-dependent heat-shock response and subsequent induction of Hsp70 and small heat-shock-protein (sHSP) [91]. Induction of Hsp70, normally expressed at very low levels, may increase tau degradation by out-competing Hsc70 for tau binding, thus giving aberrant tau a better chance at being ubiquitinated by CHIP, an E3 ubiquitin ligase (Figure 1). Small HSP induction, particularly that of Hsp27, can also benefit neurons plagued with aberrant tau as recent work demonstrated that, when functional, Hsp27 was able to reduce tau burden in a transgenic-tau mouse model [92]. Hsp27 has also been shown to have neuroprotective effects against other drivers of AD pathology [93]. And third, many kinases that regulate tau are also clients of Hsp90. Indeed, it was recently shown that Hsp90 inhibition particularly impacts kinase stability [94]. Hsp90’s role as a signaling node, connecting many regulatory pathways and regulating disease-specific processes, adds to its potential as a therapeutic target for inhibition in tau-based neurodegeneration: not only can the hyperphosphorylated tau be reduced but the kinases contributing to its persistent hyperphosphorylation can also be down-regulated. Thus, Hsp90 inhibition potential has greater efficacy than therapeutics that target single players in the processing pathways of these pathogenic proteins. When considered with decreased proteasome function or defective autophagy, as is suspected to occur in AD and many other neurodegenerative conditions [95-98], Hsp90 inhibition could reduce the tau burden from an overburdened and dysregulated regulatory system. As will be discussed later, similar regulatory systems may control the fates of other neurodegenerative proteins.

While these studies demonstrate the molecular effects of Hsp90 inhibition on tau degradation, validation of this therapeutic approach awaits the results of experiments designed to test these inhibitor’s abilities to prevent and/or improve cognitive and behavioural deficits associated with tauopathies. Exactly how tau reduction will impact these functions is still unclear. Much will depend on the timing and duration of therapeutic intervention and how much neuronal damage has already occurred. These studies also highlight the critical role of Hsp90 in facilitating neurodegenerative phenotypes and they demonstrate how Hsp90 inhibition affects select client proteins through multiple pathways.

### Therapeutic modulation of Hsp70 in AD

Controlling the expression and activity of Hsp70 shows some promise in vitro and in cell models of tau-based neurodegeneration. Work from Dickey’s group has identified both activators and inhibitors of Hsp70 activity [65,69,99-101]. Activation of Hsp70 increased tau stability, possibly by passing tau through an unproductive folding cycle. Conversely, Hsp70 inhibition reduced tau levels in cell-based models, perhaps by locking client tau and Hsp70 together, resulting in their ubiquitination and degradation [99]. They hypothesized that Hsp90 inhibition and the subsequent induction of Hsp70, followed by Hsp70 inhibition might be a potent strategy at reducing aberrant tau levels [65,99].

It is important to note, however, that Hsp70 proteins are well-characterized protectors against stress-induced apoptosis [102-104]. Hsp70 and Hsc70 silencing seems to sensitize cancer cells to Hsp90 inhibition by reducing pro-apoptotic signaling [105]. While apoptotic outcomes are desirable in cancer, it seems unlikely that widespread neural apoptosis would benefit neurodegenerative diseases like AD. Indeed, Hsp70 limits apoptosis in neurons expressing mutant androgen receptor, the protein responsible for SBMA [106]. Thus strategies that inhibit Hsp70, especially after Hsp90 inhibition, may increase the risk of neural apoptosis. It will be important for future studies to determine the possible therapeutic risks and benefits of Hsp70 inhibition in patients with tauopathy.

## Chaperones in Parkinson’s Disease

### Alpha-synuclein, LRRK2 and the chaperone network

Alpha-synuclein is highly abundant in the pre-synaptic terminals of brain tissue [107]. It comprises the primary fibrillar component of Lewy body inclusions commonly found in Parkinson’s disease (PD) patient brains [108] and its transgenic overexpression in mice facilitates neurodegeneration [109-112]. Postmortem analysis revealed that Hsp90, Hsc70 and Hsp40 co-localized with α-synuclein in Lewy bodies of α-synucleinopathy patients [113] and that CHIP and Hsp70 co-localized with α-synuclein in Lewy bodies of DLB patients [114]. Like tau, α-synuclein is an intrinsically disordered protein and can form oligomeric species that differ in size and shape [115] some of which can be toxic [116-118]. Mutations that promote α-synuclein aggregation [119-124] and α-synuclein duplications cause familial PD [125,126]. Further, Hsp90, Hsp70 and CHIP interact with α-synuclein. Hsp90 can influence α-synuclein aggregation and vesicle binding; as was demonstrated in *in vitro* studies where Hsp90 binding to α-synuclein both abolished the ability of α-synuclein to bind to small unilamellar vesicles and promoted fibril formation in an ATP-dependent manner via oligomeric intermediates [127]. Indirect data support the notion that Hsp70 likely binds monomeric forms of soluble α-synuclein: following Hsp70 depletion, α-synuclein reactions resumed at a rate similar to the initial monomer-containing reactions [128]. CHIP, by co-immunoprecipitation, was also shown to interact with α-synuclein and Hsp70 in transfected H4 cells [129]. CHIP overexpression also reduced high molecular weight α-synuclein oligomers as well as mediated α-synuclein degradation via both proteasomal and lysosomal pathways in H4 cells [129]. While the field continues to explore possible mechanisms of α-synuclein-induced toxicity, in vitro studies suggest that Hsp90, Hsp70 and CHIP likely interact in a dynamic process to regulate α-synuclein fibril assembly and degradation.

Intraneuronal inclusions found in Parkinson’s patients can also contain leucine-repeat- rich kinase 2 (LRRK2). The LRRK2G2019S substitution is the most common sporadic and inherited PD-causing mutation and is associated with dominantly inherited PD [130-133]. Mechanisms similar to, yet distinct from, α-synuclein aggregation may govern the function and stability of LRRK2 in Parkinson’s disease. As with α-synuclein, Hsp90, Hsp70 and CHIP also interact with LRRK2. Several LRRK2 proteomic studies have independently verified that LRRK2 binds to Hsp90 [134-137]. Lichtenberg et al. found that Hsp70 overexpression in COS-7 cells decreased LRRK2 aggregation [138] but did not influence levels of soluble LRRK2. LRRK2 expression in Zebrafish embryos increased GFP ubiquitination. The authors suggested that LRRK2 is more accessible in the presence of Hsp70 and that certain LRRK2 species, such as LRRK2 mono- or oligomers may mediate the accumulation of other proteins. CHIP can bind to multiple LRRK2 domains and promote LRRK2 degradation in HEK293 cells [134]. Further, Hsp90 and CHIP activity levels are determinants of LRRK2-mediated toxicity [134,139]. CHIP overexpression and increased LRRK2 degradation rates in HEK293 cells [134] and also reduced LRRK2-mediated toxicity in SH-SY5Y cells and HeLa cells.

### Hsp90 inhibition in parkinson’s disease models

Multiple in vitro studies determined that small molecule Hsp90 inhibitors have the potential to treat Parkinson’s disease. In human H4 neuroglioma cells, Hsp90 inhibitors reduced α-synuclein-induced toxicity [140,141]. Hsp90 inhibitors also rescued the axonal growth retardation caused by LRRK2G2019S in primary mouse cultured cortical neurons [142].

Additionally, Hsp90 inhibition reduced the toxicity mediated by LRRK2G2019S and LRRK2R1441C in transfected HeLa cells [139].

*In vivo* studies in PD models have been limited, perhaps largely because researchers are still in the process of developing and refining effective brain-permeable Hsp90 inhibitors. Hsp90 inhibitors prevented α-synuclein oligomer formation in culture [140] but the in vivo effects on α-synuclein have not been reported. Hsp90 inhibition rescued α-synuclein induced toxicity in Drosophila [84]. Further, a MPTP-damage mouse model of PD also revealed neuroprotective effects of Hsp90 inhibition [143].

While Hsp90 inhibitors have shown great promise for reducing protein levels, it is important to note that a number of PD-causing mutations are the result of inactive or under- functioning domains. For example, PTEN-induced kinase 1 (Pink1) PD-causing mutations likely reduce Pink1 activity [144-146] are linked to familial PD and Pink1 haplo-insufficiency is linked to idiopathic PD [147-149]. Pink-1 is an Hsp90 client [150,151] and its function may rely partly on its interaction with Hsp90. Additionally, loss-of-function mutations in parkin, an E3 ubiquitin ligase similar to CHIP, are causative factors in some familial PD [152]. If loss-of-function proteins are drivers of α-synucleinopathies like Parkinson’s disease, the potential in vivo benefits of Hsp90 inhibitors still exist as Hsp90 inhibition drives pro-clearance pathways regardless of client functionality.

In addition to directly modulating Hsp90 clients, Hsp90 inhibitors may exert indirect protective effects resulting from HSF-1 activation and subsequent Hsp70 induction. In Drosophila and yeast models of PD, directed expression of Hsp70 or heat shock (presumably through Hsp70 induction) limited α-synuclein cytotoxicity [84,153]. Hsp70 can also prevent pre- fibrillar α-synuclein formation [128,154] and reduce LRRK2 aggregation [138].

## Chaperones and Amyotrophic Lateral Sclerosis ALS

Amyotrophic lateral sclerosis (ALS) is a fatal degenerative disease of upper and lower motor neurons and the average lifespan for patients with ALS is only 3-5 years after diagnosis. Currently, over 250 mutations in 11 genes have been linked to a spectrum of familial (fALS) and sporadic forms of ALS [155,156]. Once thought to be more than 95% sporadic, it has been argued that many fALS cases are likely misreported as sporadic and should more properly be referred to as isolated ALS (iALS) [155]. Recent molecular analyses indicate a majority of familial ALS is caused by mutations in one of three proteins: the Cu/Zn superoxide dismutase SOD1 or one of two RNA-processing proteins, TAR-DNA-binding protein-43 (TDP-43) or fused- in-sarcoma (FUS), the last two of which are also linked to FTLD [157]. As with tauopathies and Parkinson’s disease, evidence supports a chaperone-mediated hypothesis of disease for much of ALS. Most importantly, therapeutic strategies that inhibit Hsp90 and increase Hsp70 activity show promise in some cellular and animal ALS models.

## SOD1

Since mutant SOD1 was first associated with insoluble intracellular inclusions from familial ALS (fALS) patients [158], it is now estimated that 20% of fALS is associated with SOD1 mutations [159]. SOD1 normally protects cells from oxidative damage, dismutating the free radical superoxide to oxygen and hydrogen peroxide. Disease-related SOD1 mutations are thought to confer a gain-of-function, as mice expressing dismutase-inactivated SOD1 develop ALS [160]. While many theories have been proposed to explain SOD1-mediated neurodegeneration, it likely arises from disruption of multiple cellular systems including metabolic signaling and metal homeostasis [161,162]. Interestingly, the contribution of aberrant SOD1 to disease depends on the cell type expressing SOD1: neuronal SOD1 influences disease genesis while astrocyte and microglial expression can accelerate disease progression [163-166].

Similar to aberrant proteins in other neurodegenerative diseases, mutant SOD1 is a client of the Hsp70/Hsp90 chaperone network [167], and its proteasomal degradation is largely regulated by the ubiquitin ligase CHIP [167-169]. Ubiquitinated SOD1 is a primary component in the intraneuronal aggregates in some ALS [158]. Hsc70 is commonly found associated with these aggregates [170,171]. This might suggest the possibility of a situation similar to that of tau and Hsc70 [64]: Hsc70 might protect SOD1 from proteasomal degradation and facilitate its accumulation [64], while Hsp70 binding might favor SOD1 ubiquitination and degradation. In cultured motor neurons expressing mutant SOD1, increasing levels of Hsp70 decreased aggregation of SOD1 and attenuated toxicity [172,173]. Raising Hsp70 levels by overexpression [174], increased HSF-1 activity or Hsp90 inhibition with 17-AAG was cytoprotective and decreased mutant SOD1 levels in primary motor neuron cultures [175]. Conversely, Liu et al. reported no benefit from over-expression of Hsp70 in mutant SOD1 mice [176]. Taken together, these results might suggest that in cellular models of SOD1-based ALS, increasing Hsp70 activity is sufficient to decrease SOD1 aggregation and limit neurotoxicity. However, in vivo models of ALS appear to benefit more from the induction of a generalized Hsf1-dependent heat-shock response. The compound arimoclomol, a “co-inducer” of the heat- shock response, reduced aggregated SOD1, delayed disease progression and increased lifespan in mutant SOD1 mice [177,178]. Recent Phase II and Phase III clinical trials with arimoclomol in SOD1-fALS patients ended and the results are expected in the near future.

## TDP-43 and FUS

A break-through in our understanding of ALS began last decade when insoluble, ubiquitinated RNA-binding protein TDP-43 was discovered in patients with ALS and FTLD [179,180]. Soon thereafter, mutated TDP-43 was linked to both fALS and iALS [181-183]. After mutations in a structurally similar RNA-binding protein, FUS, were also linked causatively to fALS [184,185], RNA processing gained wider attention in neurodegenerative research. Many recent studies implicate both loss- and gain-of-function toxic effects in TDP-43/FUS pathogenicity. TDP-43 normally regulates expression and splicing of mRNA transcripts longer than 1000 kilobases [186], including transcripts for proteins involved in synaptic function like the N-methyl-D-aspartate (NMDA) receptor and transcripts for proteins implicated in neurodegeneration like tau, parkin, huntingtin, the ataxin proteins and FUS [186-188]. TDP-43 and FUS are binding partners that may act in concert to promote toxicity. ALS-linked TDP-43 mutations increase protein stability and increase its association with FUS [189]. A complex of the two proteins inhibits cell growth in yeast [190] and impacts movement and lifespan in Drosophila [191], effects possibly mediated by co-regulation of histone deacetylase 6 (HDAC6) mRNA [192].

TDP-43 disease pathology appears to be controlled by the Hsp90 chaperone network. In a Drosophila model of ALS, 17-AAG treatment decreased and redistributed TDP-43 and decreased levels of its toxic proteolytic product TDP-25 [193]. Indeed, neurotoxicity of the TDP- 43A315T mutant in Drosophila was mitigated by Hsp70 over-expression [194]. These results suggest a regulatory role for Hsp70 and Hsp90 in TDP-43 proteostasis and pathogenicity either directly or indirectly. While much remains to be elucidated about the nature and regulation of these disease-causing proteins, these studies highlight the importance of Hsp90 and its extensive co-chaperone machinery in ALS disease processes.

## Chaperones and Polyglutamine Diseases

Some of the earliest clues to chaperone involvement in neurodegeneration came when the Hsp90 co-chaperones Hsp70 and Hsp40 were linked to polyglutamine-expansion (polyQ) diseases. These neurodegenerative diseases involve cytotoxic accumulation of a mutated protein that contains expanded tracts of glutamine residues. Increased CAG nucleotide repeats forming a series of uninterrupted glutamines disrupts native protein structure and promotes aggregate formation.

Polyglutamine expansion of ataxin proteins causes spinocerebellar ataxia (SCA), a spectrum of progressive neurodegenerative diseases currently attributed to mutations in over 29 genes [195]. Much evidence suggests the Hsp90 chaperone network regulates mutant ataxin biology. Early results from cell models demonstrated Hsp70 and Hsp40 prevented in situ aggregation of mutant ataxin-1 (SCA1) [196]. Overexpression of Hsp70 was also shown to provide neuroprotection in two in vivo models of CAG-expansion ataxia [197,198]. The proteasomal degradation of both ataxin-1 and ataxin-3 is controlled by CHIP-ubiquitination [199-202], an interaction mediated under stress conditions by Hsp70 [199]. Samples taken from patients with spinocerebellar ataxia type-7, another polyQ disease brought on by CAG repeats, demonstrated decreased expression of two major heat shock proteins, Hsp70 and Hsp27 [203]. Other groups have also demonstrated that expression of Hsp70 can be beneficial, or lack-there- of detrimental, in select polyQ ataxin disorders [204,205]. The observed neuroprotection combined with the genetic suppression suggests that induction of Hsp70 could be beneficial in cases of polyQ expanded ataxins.

Chaperone modulation also limited aggregation of mutant polyQ androgen receptor in cellular models of spinal and bulbar muscular atrophy (SBMA) [106,206], a slowly-progressive X-linked neurodegenerative disease. In 2005, Waza et al. provided the first evidence of high- ATP-affinity Hsp90 in neurodegenerative disease, when they showed 17-AAG bound to a complex of Hsp90 and mutant androgen receptor in both cellular and mouse SBMA models [207]. Additionally, treatment with the orally-bioavailable GA derivative 17- (dimethylaminoethylamino)-17-demethoxygeldanamycin (17-DMAG) limited cellular damage and improved motor function in SBMA mice [208]. It is important to note that Hsp90 inhibition caused specific degradation of mutant androgen receptor and did not affect levels of wild-type receptor. Perhaps not surprisingly, Hsp90 inhibition prevented aggregation of mutant androgen receptor in a cellular model that lacked Hsf1 and could not initiate a heat-shock response upon Hsp90 induction [209]. However, in a SBMA mouse model, overexpression of Hsp70 was able to improve the phenotype by reducing the levels of the mutant AR [210]. This finding suggests that Hsp90 inhibitors would be able to improve the health of neurons expressing mutant AR by both Hsp90 inhibition and concurrent Hsp70 induction.

Perhaps the best-known polyQ disease is Huntington’s disease, characterized by the aggregation of the multi-functional huntingtin protein. Similar to the other polyglutamine- expansion diseases, increasing cellular Hsp70 and Hsp40 levels limited abnormal huntingtin aggregation [211]. Inhibition of Hsp90 and Hsp70 also reduced aggregation of huntingtin [83,212,213], which was recently confirmed as an Hsp90 client [214]. Interestingly, huntingtin degradation is also regulated by CHIP [215,216]. Increasing the levels of CHIP reduced both huntingtin aggregation and aggregation-dependent apoptosis [201]. Similar to many other chaperone-dependent neurodegenerative diseases we’ve discussed, Hsc70 is commonly observed associated with insoluble huntingtin cellular and brain aggregates [217]. In a *Saccharomyces cerevisae* model of huntingtin aggregation other heat shock proteins including Hsp26 and Hsp104 interacted with huntigntin [218]. Additionally, BAG1, the same protein that influences tau fate through Hsc70 binding, may be playing a similar role in determining the aggregation fate of huntingtin [219-224].

All of these results suggest that chaperones play critical roles in facilitating the accumulation of mutated proteins in polyglutamine expansion diseases. The possible roles of Hsp70 and Hsp90 in this disease system are strikingly similar to their suspected roles in facilitating tauopathy and other neurodegenerative diseases. However, while Hsp90 inhibition and increased Hsp70 expression have shown promise in alleviating toxic pathology of the polyglutamine diseases in both cellular and animal models, their effectiveness as therapeutic strategies awaits validation in clinical trials.

## Conclusions

As reviewed here, the Hsp90 chaperone network likely facilitates many proteinopathic neurodegenerative diseases. Basal disease conditions dominated by constitutive Hsc70 expression favour the binding of aberrant clients to Hsc70. This client/Hsc70 complex then presents the client to Hsp90 for (presumably) unsuccessful folding. Since Hsc70 is commonly associated with insoluble client aggregates, we can surmise that, during disease conditions, Hsc70 binding to aberrant clients favours client accumulation and aggregation. Upon Hsp90 inhibition, however, two important changes take place. First, the folding pathway of Hsp90 is eliminated as an option, shunting the flow of cycling clients toward degradation pathways. Second, Hsp70 expression is greatly increased with the induced heat-shock response. Experimental evidence shows that Hsp70 binding results in aberrant client degradation, most likely by increasing the possibility that the Hsp70-client complex will be ubiquitinated by CHIP and eventually degraded. While increasing Hsp70 expression alone can decrease levels of aberrant client proteins, results from both cell and animal models of neurodegeneration suggest that the greatest benefit comes from Hsp90 inhibition and the combined actions of stopping client folding and increasing Hsp70 expression. Thus, pharmacologic inhibition of Hsp90 and induction of a neuroprotective heat-shock response have a two-pronged effect on aggregating neurodegenerative clients, in that the inhibition breaks the “non-functional folding cycle” driven by Hsp90 and the subsequent induction of HSPs increases the presence of the pro-degradation Hsp70 (Figures 1 and 2).

The role of the Hsp90 chaperone network machinery in the regulation of aberrant proteins makes it a prime therapeutic target in treating the spectrum of neurodegenerative diseases (Table 1). While Hsp90 inhibitors clearly show therapeutic potential in multiple animal models expressing mutant aggregating proteins, it remains uncertain whether they will prove similarly successful in the larger number of patients with idiopathic disease. Despite this, mounting evidence suggests that most of these neurodegenerative models also benefit from induction of the heat-shock response and/or increased expression of Hsp70 and the subsequent degradation of problem proteins. This offers some hope that these processes may be generalized to many proteinopathic neurodegenerative diseases. Much work remains to fully elucidate the roles of Hsp90 network chaperones in neurodegenerative disease. Experimental results from the last decade speak to the potential of chaperone manipulation, specifically Hsp90 inhibition and up-regulation of Hsp70, as promising therapeutic paradigms that may be applicable across a broad range of neurodegenerative disease.

## Figures and Tables

**Table 1:** Shows Inhibition of Hsp90 and Hsp 70 which generates two distinct mechanisms both of which result in client protein degradation and enhanced cell survival.

Protein	Associated Neurodegenerative disease	Benefit from Hsp70 elevation *in vitro*?	Benefit from Hsp70 elevation *in vivo*?	Benefit from Hsp90 inhibition *in vitro*?	Benefit from Hsp90 inhibition *in vivo*?
**Tau**	Tauopathies (AD, CBD, PSP, FTDP-17)	Yes [61]	Yes [61]	Yes [62,90]	Yes [62,90]
**α-synuclein**	PD, DLB, MSA	Yes [220]	?	Yes[127,221]	Yes [140]
**LRRK2**	PD	Yes [138]	?	Yes [139]	?
**SOD1**	ALS	Yes [172-174]	No [176]	Yes [175]	?
**TDP-43**	ALS/FTD	Yes [222]	Yes [194]	Yes [223]	Yes [193]
**Huntingtin**	HD	Yes [211]	No [212]	Yes [83]	Yes [212]
**Androgen receptor**	SBMA	Yes [106,206]	Yes [210,224]	Yes[207,209]	Yes [207,208]
**Ataxins**	SCA	Yes [196]	Yes [197,198]	?	?

## References

[1] Neckers L, Ivy SP (2003). Heat shock protein 90. Curr Opin Oncol.

[2] Sõti C, Nagy E, Giricz Z, Vígh L, Csermely P (2005). Heat shock proteins as emerging therapeutic targets. Br J Pharmacol.

[3] Nollen EA, Morimoto RI (2002). Chaperoning signaling pathways: molecular chaperones as stress-sensing ‘heat shock’ proteins. J Cell Sci.

[4] Whitesell L, Mimnaugh EG, De Costa B, Myers CE, Neckers LM (1994). Inhibition of heat shock protein hsp90-pp60v-src heteroprotein complex formation by benzoquinone ansamycins: Essential role for stress proteins in oncogenic transformation. Proc Natl Acad Sci U S A.

[5] Stancato LF, Silverstein AM, Owens-Grillo JK, Chow YH, Jove R (1997). The hsp90-binding antibiotic geldanamycin decreases Raf levels and epidermal growth factor signaling without disrupting formation of signaling complexes or reducing the specific enzymatic activity of Raf kinase. J Biol Chem.

[6] Schulte TW, Neckers LM (1998). The benzoquinone ansamycin 17-allylamino-17-demethoxygeldanamycin binds to HSP90 and shares important biologic activities with geldanamycin. Cancer Chemother Pharmacol.

[7] Stebbins CE, Russo AA, Schneider C, Rosen N, Hartl FU (1997). Crystal structure of an Hsp90-geldanamycin complex: targeting of a protein chaperone by an antitumor agent. Cell.

[8] Kamal A, Thao L, Sensintaffar J, Zhang L, Boehm MF (2003). A high-affinity conformation of Hsp90 confers tumour selectivity on Hsp90 inhibitors. Nature.

[9] Moulick K, Ahn JH, Zong H, Rodina A, Cerchietti L (2011). Affinity-based proteomics reveal cancer-specific networks coordinated by Hsp90. Nat Chem Biol.

[10] Mollapour M, Neckers L (2012). Post-translational modifications of Hsp90 and their contributions to chaperone regulation. Biochim Biophys Acta.

[11] Ikenaka K, Katsuno M, Kawai K, Ishigaki S, Tanaka F (2012). Disruption of axonal transport in motor neuron diseases. Int J Mol Sci.

[12] Rubinsztein DC, Ravikumar B, Acevedo-Arozena A, Imarisio S, O’Kane CJ (2005). Dyneins, autophagy, aggregation and neurodegeneration. Autophagy.

[13] Chevalier-Larsen E, Holzbaur EL (2006). Axonal transport and neurodegenerative disease. Biochim Biophys Acta.

[14] Himmler A (1989). Structure of the bovine tau gene: alternatively spliced transcripts generate a protein family. Mol Cell Biol.

[15] Himmler A, Drechsel D, Kirschner MW, Martin DW (1989). Tau consists of a set of proteins with repeated C-terminal microtubule-binding domains and variable N-terminal domains. Mol Cell Biol.

[16] Buée L, Delacourte A (1999). Comparative biochemistry of tau in progressive supranuclear palsy, corticobasal degeneration, FTDP-17 and Pick’s disease. Brain Pathol.

[17] Lee VM, Daughenbaugh R, Trojanowski JQ (1994). Microtubule stabilizing drugs for the treatment of Alzheimer’s disease. Neurobiol Aging.

[18] Baker M, Litvan I, Houlden H, Adamson J, Dickson D (1999). Association of an extended haplotype in the tau gene with progressive supranuclear palsy. Hum Mol Genet.

[19] de Silva R, Weiler M, Morris HR, Martin ER, Wood NW (2001). Strong association of a novel Tau promoter haplotype in progressive supranuclear palsy. Neurosci Lett.

[20] Crowther RA, Goedert M (2000). Abnormal tau-containing filaments in neurodegenerative diseases. J Struct Biol.

[21] Di Maria E, Tabaton M, Vigo T, Abbruzzese G, Bellone E (2000). Corticobasal degeneration shares a common genetic background with progressive supranuclear palsy. Ann Neurol.

[22] Lee VM, Trojanowski JQ (2001). Transgenic mouse models of tauopathies: prospects for animal models of Pick’s disease. Neurology.

[23] Hutton M, Lendon CL, Rizzu P, Baker M, Froelich S (1998). Association of missense and 5’-splice-site mutations in tau with the inherited dementia FTDP-17. Nature.

[24] Chang E, Kim S, Schafer KN, Kuret J (2011). Pseudophosphorylation of tau protein directly modulates its aggregation kinetics. Biochim Biophys Acta.

[25] Han D, Paudel HK (2009). FTDP-17 missense mutations site-specifically inhibit as well as promote dephosphorylation of microtubule-associated protein tau by protein phosphatases of HEK-293 cell extract. Neurochem Int.

[26] Nagiec EW, Sampson KE, Abraham I (2001). Mutated tau binds less avidly to microtubules than wildtype tau in living cells. J Neurosci Res.

[27] Lindwall G, Cole RD (1984). Phosphorylation affects the ability of tau protein to promote microtubule assembly. J Biol Chem.

[28] Iqbal K, Grundke-Iqbal I, Zaidi T, Merz PA, Wen GY (1986). Defective brain microtubule assembly in Alzheimer’s disease. Lancet.

[29] Han D, Qureshi HY, Lu Y, Paudel HK (2009). Familial FTDP-17 missense mutations inhibit microtubule assembly-promoting activity of tau by increasing phosphorylation at Ser202 in vitro. J Biol Chem.

[30] Grundke-Iqbal I, Iqbal K, Tung YC, Quinlan M, Wisniewski HM (1986). Abnormal phosphorylation of the microtubule-associated protein tau (tau) in Alzheimer cytoskeletal pathology. Proc Natl Acad Sci U S A.

[31] Bramblett GT, Goedert M, Jakes R, Merrick SE, Trojanowski JQ (1993). Abnormal tau phosphorylation at Ser396 in Alzheimer’s disease recapitulates development and contributes to reduced microtubule binding. Neuron.

[32] Dayanandan R, Van Slegtenhorst M, Mack TG, Ko L, Yen SH (1999). Mutations in tau reduce its microtubule binding properties in intact cells and affect its phosphorylation. FEBS Lett.

[33] von Bergen M, Barghorn S, Li L, Marx A, Biernat J (2001). Mutations of tau protein in frontotemporal dementia promote aggregation of paired helical filaments by enhancing local beta-structure. J Biol Chem.

[34] Nacharaju P, Lewis J, Easson C, Yen S, Hackett J (1999). Accelerated filament formation from tau protein with specific FTDP-17 missense mutations. FEBS Lett.

[35] Chang E, Kim S, Yin H, Nagaraja HN, Kuret J (2008). Pathogenic missense MAPT mutations differentially modulate tau aggregation propensity at nucleation and extension steps. J Neurochem.

[36] Miyasaka T, Morishima-Kawashima M, Ravid R, Kamphorst W, Nagashima K (2001). Selective deposition of mutant tau in the FTDP-17 brain affected by the P301L mutation. J Neuropathol Exp Neurol.

[37] Bugiani O, Murrell JR, Giaccone G, Hasegawa M, Ghigo G (1999). Frontotemporal dementia and corticobasal degeneration in a family with a P301S mutation in tau. J Neuropathol Exp Neurol.

[38] Allen B, Ingram E, Takao M, Smith MJ, Jakes R (2002). Abundant tau filaments and nonapoptotic neurodegeneration in transgenic mice expressing human P301S tau protein. J Neurosci.

[39] Lewis J, McGowan E, Rockwood J, Melrose H, Nacharaju P (2000). Neurofibrillary tangles, amyotrophy and progressive motor disturbance in mice expressing mutant (P301L) tau protein. Nat Genet.

[40] Scattoni ML, Gasparini L, Alleva E, Goedert M, Calamandrei G (2010). Early behavioural markers of disease in P301S tau transgenic mice. Behav Brain Res.

[41] Takeuchi H, Iba M, Inoue H, Higuchi M, Takao K (2011). P301S mutant human tau transgenic mice manifest early symptoms of human tauopathies with dementia and altered sensorimotor gating. PLoS One.

[42] Yoshiyama Y, Higuchi M, Zhang B, Huang SM, Iwata N (2007). Synapse loss and microglial activation precede tangles in a P301S tauopathy mouse model. Neuron.

[43] Gotz J, Chen F, Barmettler R, Nitsch RM (2001). Tau filament formation in transgenic mice expressing P301L tau. J Biol Chem.

[44] Sahara N, Lewis J, DeTure M, McGowan E, Dickson DW (2002). Assembly of tau in transgenic animals expressing P301L tau: alteration of phosphorylation and solubility. J Neurochem.

[45] Zhou J, Yu Q, Zou T (2008). Alternative splicing of exon 10 in the tau gene as a target for treatment of tauopathies. BMC Neurosci.

[46] Koga H, Kaushik S, Cuervo AM (2011). Protein homeostasis and aging: The importance of exquisite quality control. Ageing Res Rev.

[47] Guo JL, Lee VM (2011). Seeding of normal Tau by pathological Tau conformers drives pathogenesis of Alzheimer-like tangles. J Biol Chem.

[48] Clavaguera F, Bolmont T, Crowther RA, Abramowski D, Frank S (2009). Transmission and spreading of tauopathy in transgenic mouse brain. Nat Cell Biol.

[49] Braak H, Braak E (1991). Neuropathological stageing of Alzheimer-related changes. Acta Neuropathol.

[50] Liu L, Drouet V, Wu JW, Witter MP, Small SA (2012). Trans-synaptic spread of tau pathology in vivo. PLoS One.

[51] Lei P, Ayton S, Finkelstein DI, Spoerri L, Ciccotosto GD (2012). Tau deficiency induces parkinsonism with dementia by impairing APP-mediated iron export. Nat Med.

[52] Evans CG, Wisén S, Gestwicki JE (2006). Heat shock proteins 70 and 90 inhibit early stages of amyloid beta-(1-42) aggregation in vitro. J Biol Chem.

[53] Koren J, Jinwal UK, Lee DC, Jones JR, Shults CL (2009). Chaperone signalling complexes in Alzheimer’s disease. J Cell Mol Med.

[54] Kumar P, Ambasta RK, Veereshwarayya V, Rosen KM, Kosik KS (2007). CHIP and HSPs interact with beta-APP in a proteasome-dependent manner and influence Abeta metabolism. Hum Mol Genet.

[55] Paris D, Ganey NJ, Laporte V, Patel NS, Beaulieu-Abdelahad D (2010). Reduction of beta-amyloid pathology by celastrol in a transgenic mouse model of Alzheimer’s disease. J Neuroinflammation.

[56] Ott S, Henkel AW, Henkel MK, Redzic ZB, Kornhuber J (2011). Pre-aggregated Aβ1-42 peptide increases tau aggregation and hyperphosphorylation after short-term application. Mol Cell Biochem.

[57] Osinde M, Clavaguera F, May-Nass R, Tolnay M, Dev KK (2008). Lentivirus Tau (P301S) expression in adult amyloid precursor protein (APP)-transgenic mice leads to tangle formation. Neuropathol Appl Neurobiol.

[58] Roberson ED, Scearce-Levie K, Palop JJ, Yan F, Cheng IH (2007). Reducing endogenous tau ameliorates amyloid beta-induced deficits in an Alzheimer’s disease mouse model. Science.

[59] Götz J, Chen F, van Dorpe J, Nitsch RM (2001). Formation of neurofibrillary tangles in P301l tau transgenic mice induced by Abeta 42 fibrils. Science.

[60] Bose A, Mouton-Liger F, Paquet C, Mazot P, Vigny M (2011). Modulation of tau phosphorylation by the kinase PKR: implications in Alzheimer’s disease. Brain Pathol.

[61] Dou F, Netzer WJ, Tanemura K, Li F, Hartl FU (2003). Chaperones increase association of tau protein with microtubules. Proc Natl Acad Sci U S A.

[62] Dickey CA, Kamal A, Lundgren K, Klosak N, Bailey RM (2007). The high-affinity HSP90-CHIP complex recognizes and selectively degrades phosphorylated tau client proteins. J Clin Invest.

[63] Jinwal UK, O’Leary JC, Borysov SI, Jones JR, Li Q (2010). Hsc70 rapidly engages tau after microtubule destabilization. J Biol Chem.

[64] Elliott E, Tsvetkov P, Ginzburg I (2007). BAG-1 associates with Hsc70.Tau complex and regulates the proteasomal degradation of Tau protein. J Biol Chem.

[65] Jinwal UK, Koren J, O’Leary JC, Jones JR, Abisambra JF (2010). Hsp70 ATPase Modulators as Therapeutics for Alzheimer’s and other Neurodegenerative Diseases. Mol Cell Pharmacol.

[66] Pratt WB, Morishima Y, Peng HM, Osawa Y (2010). Proposal for a role of the Hsp90/Hsp70-based chaperone machinery in making triage decisions when proteins undergo oxidative and toxic damage. Exp Biol Med (Maywood).

[67] Shimura H, Schwartz D, Gygi SP, Kosik KS (2004). CHIP-Hsc70 complex ubiquitinates phosphorylated tau and enhances cell survival. J Biol Chem.

[68] Kundrat L, Regan L (2010). Balance between folding and degradation for Hsp90-dependent client proteins: a key role for CHIP. Biochemistry.

[69] Koren J, Jinwal UK, Jin Y, O’Leary J, Jones JR (2010). Facilitating Akt clearance via manipulation of Hsp70 activity and levels. J Biol Chem.

[70] Voss K, Combs B, Patterson KR, Binder LI, Gamblin TC (2012). Hsp70 alters tau function and aggregation in an isoform specific manner. Biochemistry.

[71] Luo W, Rodina A, Chiosis G (2008). Heat shock protein 90: translation from cancer to Alzheimer’s disease treatment?. BMC Neurosci.

[72] Miyata Y, Koren J, Kiray J, Dickey CA, Gestwicki JE (2011). Molecular chaperones and regulation of tau quality control: strategies for drug discovery in tauopathies. Future Med Chem.

[73] Salminen A, Ojala J, Kaarniranta K, Hiltunen M, Soininen H (2011). Hsp90 regulates tau pathology through co-chaperone complexes in Alzheimer’s disease. Prog Neurobiol.

[74] Jinwal UK, Koren J, Borysov SI, Schmid AB, Abisambra JF (2010). The Hsp90 cochaperone, FKBP51, increases Tau stability and polymerizes microtubules. J Neurosci.

[75] Koren J, Jinwal UK, Davey Z, Kiray J, Arulselvam K (2011). Bending tau into shape: the emerging role of peptidyl-prolyl isomerases in tauopathies. Mol Neurobiol.

[76] Taldone T, Sun W, Chiosis G (2009). Discovery and development of heat shock protein 90 inhibitors. Bioorg Med Chem.

[77] Porter JR, Fritz CC, Depew KM (2010). Discovery and development of Hsp90 inhibitors: a promising pathway for cancer therapy. Curr Opin Chem Biol.

[78] Taldone T, Chiosis G (2009). Purine-scaffold Hsp90 inhibitors. Curr Top Med Chem.

[79] Taldone T, Zatorska D, Patel PD, Zong H, Rodina A (2011). Design, synthesis, and evaluation of small molecule Hsp90 probes. Bioorg Med Chem.

[80] Janin YL (2010). ATPase inhibitors of heat-shock protein 90, second season. Drug Discov Today.

[81] Biamonte MA, Van de Water R, Arndt JW, Scannevin RH, Perret D (2010). Heat shock protein 90: inhibitors in clinical trials. J Med Chem.

[82] Caldas-Lopes E, Cerchietti L, Ahn JH, Clement CC, Robles AI (2009). Hsp90 inhibitor PU-H71, a multimodal inhibitor of malignancy, induces complete responses in triple-negative breast cancer models. Proc Natl Acad Sci U S A.

[83] Sittler A, Lurz R, Lueder G, Priller J, Lehrach H (2001). Geldanamycin activates a heat shock response and inhibits huntingtin aggregation in a cell culture model of Huntington’s disease. Hum Mol Genet.

[84] Auluck PK, Chan HY, Trojanowski JQ, Lee VM, Bonini NM (2002). Chaperone suppression of alpha-synuclein toxicity in a Drosophila model for Parkinson’s disease. Science.

[85] Dickey CA, Dunmore J, Lu B, Wang JW, Lee WC (2006). HSP induction mediates selective clearance of tau phosphorylated at proline-directed Ser/Thr sites but not KXGS (MARK) sites. FASEB J.

[86] Andorfer C, Kress Y, Espinoza M, de Silva R, Tucker KL (2003). Hyperphosphorylation and aggregation of tau in mice expressing normal human tau isoforms. J Neurochem.

[87] Noble W, Planel E, Zehr C, Olm V, Meyerson J (2005). Inhibition of glycogen synthase kinase-3 by lithium correlates with reduced tauopathy and degeneration in vivo. Proc Natl Acad Sci U S A.

[88] Jinwal UK, Trotter JH, Abisambra JF, Koren J, Lawson LY (2011). The Hsp90 kinase co-chaperone Cdc37 regulates tau stability and phosphorylation dynamics. J Biol Chem.

[89] Dickey CA, Koren J, Zhang YJ, Xu YF, Jinwal UK (2008). Akt and CHIP coregulate tau degradation through coordinated interactions. Proc Natl Acad Sci U S A.

[90] Luo W, Dou F, Rodina A, Chip S, Kim J (2007). Roles of heat-shock protein 90 in maintaining and facilitating the neurodegenerative phenotype in tauopathies. Proc Natl Acad Sci U S A.

[91] Ali A, Bharadwaj S, O’Carroll R, Ovsenek N (1998). HSP90 interacts with and regulates the activity of heat shock factor 1 in Xenopus oocytes. Mol Cell Biol.

[92] Abisambra JF, Blair LJ, Hill SE, Jones JR, Kraft C (2010). Phosphorylation dynamics regulate Hsp27-mediated rescue of neuronal plasticity deficits in tau transgenic mice. J Neurosci.

[93] Abisambra JF, Jinwal UK, Jones JR, Blair LJ, Koren J (2011). Exploiting the diversity of the heat-shock protein family for primary and secondary tauopathy therapeutics. Curr Neuropharmacol.

[94] Sharma K, Vabulas RM, Macek B, Pinkert S, Cox J (2012). Quantitative proteomics reveals that Hsp90 inhibition preferentially targets kinases and the DNA damage response. Mol Cell Proteomics.

[95] Cuervo AM, Wong ES, Martinez-Vicente M (2010). Protein degradation, aggregation, and misfolding. Mov Disord.

[96] Lee S, Sato Y, Nixon RA (2011). Lysosomal proteolysis inhibition selectively disrupts axonal transport of degradative organelles and causes an Alzheimer’s-like axonal dystrophy. J Neurosci.

[97] Nixon RA (2007). Autophagy, amyloidogenesis and Alzheimer disease. J Cell Sci.

[98] Yu WH, Cuervo AM, Kumar A, Peterhoff CM, Schmidt SD (2005). Macroautophagy--a novel Beta-amyloid peptide-generating pathway activated in Alzheimer’s disease. J Cell Biol.

[99] Jinwal UK, Miyata Y, Koren J, Jones JR, Trotter JH (2009). Chemical manipulation of hsp70 ATPase activity regulates tau stability. J Neurosci.

[100] Jones JR, Lebar MD, Jinwal UK, Abisambra JF, Koren J (2011). The diarylheptanoid (+)-aR,11S-myricanol and two flavones from bayberry (Myrica cerifera) destabilize the microtubule-associated protein tau. J Nat Prod.

[101] Koren J, Miyata Y, Kiray J, O’Leary JC, Nguyen L (2012). Rhodacyanine derivative selectively targets cancer cells and overcomes tamoxifen resistance. PLoS One.

[102] Wei YQ, Zhao X, Kariya Y, Teshigawara K, Uchida A (1995). Inhibition of proliferation and induction of apoptosis by abrogation of heat-shock protein (HSP) 70 expression in tumor cells. Cancer Immunol Immunother.

[103] Dix DJ, Allen JW, Collins BW, Mori C, Nakamura N (1996). Targeted gene disruption of Hsp70-2 results in failed meiosis, germ cell apoptosis, and male infertility. Proc Natl Acad Sci U S A.

[104] Mosser DD, Caron AW, Bourget L, Meriin AB, Sherman MY (2000). The chaperone function of hsp70 is required for protection against stress-induced apoptosis. Mol Cell Biol.

[105] Powers MV, Clarke PA, Workman P (2008). Dual targeting of HSC70 and HSP72 inhibits HSP90 function and induces tumor-specific apoptosis. Cancer Cell.

[106] Kobayashi Y, Kume A, Li M, Doyu M, Hata M (2000). Chaperones Hsp70 and Hsp40 suppress aggregate formation and apoptosis in cultured neuronal cells expressing truncated androgen receptor protein with expanded polyglutamine tract. J Biol Chem.

[107] George JM (2002). The synucleins. Genome Biol.

[108] Irizarry MC, Growdon W, Gomez-Isla T, Newell K, George JM (1998). Nigral and cortical Lewy bodies and dystrophic nigral neurites in Parkinson’s disease and cortical Lewy body disease contain alpha-synuclein immunoreactivity. J Neuropathol Exp Neurol.

[109] Springer W, Kahle PJ (2006). Mechanisms and models of alpha-synuclein-related neurodegeneration. Curr Neurol Neurosci Rep.

[110] Masliah E, Rockenstein E, Veinbergs I, Mallory M, Hashimoto M (2000). Dopaminergic loss and inclusion body formation in alpha-synuclein mice: implications for neurodegenerative disorders. Science.

[111] Lee MK, Stirling W, Xu Y, Xu X, Qui D (2002). Human alpha-synuclein-harboring familial Parkinson’s disease-linked Ala-53 --> Thr mutation causes neurodegenerative disease with alpha-synuclein aggregation in transgenic mice. Proc Natl Acad Sci U S A.

[112] Fernagut PO, Chesselet MF (2004). Alpha-synuclein and transgenic mouse models. Neurobiol Dis.

[113] Uryu K, Richter-Landsberg C, Welch W, Sun E, Goldbaum O (2006). Convergence of heat shock protein 90 with ubiquitin in filamentous alpha-synuclein inclusions of alpha-synucleinopathies. Am J Pathol.

[114] Shin Y, Klucken J, Patterson C, Hyman BT, McLean PJ (2005). The co-chaperone carboxyl terminus of Hsp70-interacting protein (CHIP) mediates alpha-synuclein degradation decisions between proteasomal and lysosomal pathways. J Biol Chem.

[115] Uversky VN (2007). Neuropathology, biochemistry, and biophysics of alpha-synuclein aggregation. J Neurochem.

[116] Wright JA, Wang X, Brown DR (2009). Unique copper-induced oligomers mediate alpha-synuclein toxicity. FASEB J.

[117] Pountney DL, Voelcker NH, Gai WP (2005). Annular alpha-synuclein oligomers are potentially toxic agents in alpha-synucleinopathy. Hypothesis. Neurotox Res.

[118] Lashuel HA, Petre BM, Wall J, Simon M, Nowak RJ (2002). Alpha-synuclein, especially the Parkinson’s disease-associated mutants, forms pore-like annular and tubular protofibrils. J Mol Biol.

[119] Spillantini MG, Schmidt ML, Lee VM, Trojanowski JQ, Jakes R (1997). Alpha-synuclein in Lewy bodies. Nature.

[120] Polymeropoulos MH, Lavedan C, Leroy E, Ide SE, Dehejia A (1997). Mutation in the alpha-synuclein gene identified in families with Parkinson’s disease. Science.

[121] Narhi L, Wood SJ, Steavenson S, Jiang Y, Wu GM (1999). Both familial Parkinson’s disease mutations accelerate alpha-synuclein aggregation. J Biol Chem.

[122] Krüger R, Kuhn W, Müller T, Woitalla D, Graeber M (1998). Ala30Pro mutation in the gene encoding alpha-synuclein in Parkinson’s disease. Nat Genet.

[123] Giasson BI, Uryu K, Trojanowski JQ, Lee VM (1999). Mutant and wild type human alpha-synucleins assemble into elongated filaments with distinct morphologies in vitro. J Biol Chem.

[124] El-Agnaf OM, Jakes R, Curran MD, Wallace A (1998). Effects of the mutations Ala30 to Pro and Ala53 to Thr on the physical and morphological properties of alpha-synuclein protein implicated in Parkinson’s disease. FEBS Lett.

[125] Ibáñez P, Bonnet AM, Débarges B, Lohmann E, Tison F (2004). Causal relation between alpha-synuclein gene duplication and familial Parkinson’s disease. Lancet.

[126] Chartier-Harlin MC, Kachergus J, Roumier C, Mouroux V, Douay X (2004). Alpha-synuclein locus duplication as a cause of familial Parkinson’s disease. Lancet.

[127] Falsone SF, Kungl AJ, Rek A, Cappai R, Zangger K (2009). The molecular chaperone Hsp90 modulates intermediate steps of amyloid assembly of the Parkinson-related protein alpha-synuclein. J Biol Chem.

[128] Luk KC, Mills IP, Trojanowski JQ, Lee VM (2008). Interactions between Hsp70 and the hydrophobic core of alpha-synuclein inhibit fibril assembly. Biochemistry.

[129] Tetzlaff JE, Putcha P, Outeiro TF, Ivanov A, Berezovska O (2008). CHIP targets toxic alpha-Synuclein oligomers for degradation. J Biol Chem.

[130] Lesage S, Ibanez P, Lohmann E, Pollak P, Tison F (2005). G2019S LRRK2 mutation in French and North African families with Parkinson’s disease. Ann Neurol.

[131] Healy DG, Falchi M, O’Sullivan SS, Bonifati V, Durr A (2008). Phenotype, genotype, and worldwide genetic penetrance of LRRK2-associated Parkinson’s disease: a case-control study. Lancet Neurol.

[132] Goldwurm S, Di Fonzo A, Simons EJ, Rohé CF, Zini M (2005). The G6055A (G2019S) mutation in LRRK2 is frequent in both early and late onset Parkinson’s disease and originates from a common ancestor. J Med Genet.

[133] Bras JM, Guerreiro RJ, Ribeiro MH, Januario C, Morgadinho A (2005). G2019S dardarin substitution is a common cause of Parkinson’s disease in a Portuguese cohort. Mov Disord.

[134] Ding X, Goldberg MS (2009). Regulation of LRRK2 stability by the E3 ubiquitin ligase CHIP. PLoS One.

[135] Gloeckner CJ, Kinkl N, Schumacher A, Braun RJ, O’Neill E (2006). The Parkinson disease causing LRRK2 mutation I2020T is associated with increased kinase activity. Hum Mol Genet.

[136] Meixner A, Boldt K, Van Troys M, Askenazi M, Gloeckner CJ (2011). A QUICK screen for Lrrk2 interaction partners--leucine-rich repeat kinase 2 is involved in actin cytoskeleton dynamics. Mol Cell Proteomics.

[137] Zach S, Felk S, Gillardon F (2010). Signal transduction protein array analysis links LRRK2 to Ste20 kinases and PKC zeta that modulate neuronal plasticity. PLoS One.

[138] Lichtenberg M, Mansilla A, Zecchini VR, Fleming A, Rubinsztein DC (2011). The Parkinson’s disease protein LRRK2 impairs proteasome substrate clearance without affecting proteasome catalytic activity. Cell Death Dis.

[139] Ko HS, Bailey R, Smith WW, Liu Z, Shin JH (2009). CHIP regulates leucine-rich repeat kinase-2 ubiquitination, degradation, and toxicity. Proc Natl Acad Sci U S A.

[140] Putcha P, Danzer KM, Kranich LR, Scott A, Silinski M (2010). Brain-permeable small-molecule inhibitors of Hsp90 prevent alpha-synuclein oligomer formation and rescue alpha-synuclein-induced toxicity. J Pharmacol Exp Ther.

[141] McLean PJ, Klucken J, Shin Y, Hyman BT (2004). Geldanamycin induces Hsp70 and prevents alpha-synuclein aggregation and toxicity in vitro. Biochem Biophys Res Commun.

[142] Wang L, Xie C, Greggio E, Parisiadou L, Shim H (2008). The chaperone activity of heat shock protein 90 is critical for maintaining the stability of leucine-rich repeat kinase 2. J Neurosci.

[143] Shen HY, He JC, Wang Y, Huang QY, Chen JF (2005). Geldanamycin induces heat shock protein 70 and protects against MPTP-induced dopaminergic neurotoxicity in mice. J Biol Chem.

[144] Sim CH, Lio DS, Mok SS, Masters CL, Hill AF (2006). C-terminal truncation and Parkinson’s disease-associated mutations down-regulate the protein serine/threonine kinase activity of PTEN-induced kinase-1. Hum Mol Genet.

[145] Silvestri L, Caputo V, Bellacchio E, Atorino L, Dallapiccola B (2005). Mitochondrial import and enzymatic activity of PINK1 mutants associated to recessive parkinsonism. Hum Mol Genet.

[146] Beilina A, Van Der Brug M, Ahmad R, Kesavapany S, Miller DW (2005). Mutations in PTEN-induced putative kinase 1 associated with recessive parkinsonism have differential effects on protein stability. Proc Natl Acad Sci U S A.

[147] Gispert S, Del Turco D, Garrett L, Chen A, Bernard DJ (2003). Transgenic mice expressing mutant A53T human alpha-synuclein show neuronal dysfunction in the absence of aggregate formation. Mol Cell Neurosci.

[148] Klein C, Schlossmacher MG (2006). The genetics of Parkinson disease: Implications for neurological care. Nat Clin Pract Neurol.

[149] Valente EM, Abou-Sleiman PM, Caputo V, Muqit MM, Harvey K (2004). Hereditary early-onset Parkinson’s disease caused by mutations in PINK1. Science.

[150] Weihofen A, Ostaszewski B, Minami Y, Selkoe DJ (2008). Pink1 Parkinson mutations, the Cdc37/Hsp90 chaperones and Parkin all influence the maturation or subcellular distribution of Pink1. Hum Mol Genet.

[151] Moriwaki Y, Kim YJ, Ido Y, Misawa H, Kawashima K (2008). L347P PINK1 mutant that fails to bind to Hsp90/Cdc37 chaperones is rapidly degraded in a proteasome-dependent manner. Neurosci Res.

[152] Henn IH, Gostner JM, Lackner P, Tatzelt J, Winklhofer KF (2005). Pathogenic mutations inactivate parkin by distinct mechanisms. J Neurochem.

[153] Flower TR, Chesnokova LS, Froelich CA, Dixon C, Witt SN (2005). Heat shock prevents alpha-synuclein-induced apoptosis in a yeast model of Parkinson’s disease. J Mol Biol.

[154] Huang C, Cheng H, Hao S, Zhou H, Zhang X (2006). Heat shock protein 70 inhibits alpha-synuclein fibril formation via interactions with diverse intermediates. J Mol Biol.

[155] Andersen PM, Al-Chalabi A (2011). Clinical genetics of amyotrophic lateral sclerosis: what do we really know?. Nat Rev Neurol.

[156] Da Cruz S, Cleveland DW (2011). Understanding the role of TDP-43 and FUS/TLS in ALS and beyond. Curr Opin Neurobiol.

[157] Mackenzie IR, Rademakers R, Neumann M (2010). TDP-43 and FUS in amyotrophic lateral sclerosis and frontotemporal dementia. Lancet Neurol.

[158] Rosen DR, Siddique T, Patterson D, Figlewicz DA, Sapp P (1993). Mutations in Cu/Zn superoxide dismutase gene are associated with familial amyotrophic lateral sclerosis. Nature.

[159] Joyce PI, Fratta P, Fisher EM, Acevedo-Arozena A (2011). SOD1 and TDP-43 animal models of amyotrophic lateral sclerosis: recent advances in understanding disease toward the development of clinical treatments. Mamm Genome.

[160] Bruijn LI, Becher MW, Lee MK, Anderson KL, Jenkins NA (1997). ALS-linked SOD1 mutant G85R mediates damage to astrocytes and promotes rapidly progressive disease with SOD1-containing inclusions. Neuron.

[161] Sehati S, Clement MH, Martins J, Xu L, Longo VD (2011). Metabolic alterations in yeast lacking copper-zinc superoxide dismutase. Free Radic Biol Med.

[162] Strain J, Lorenz CR, Bode J, Garland S, Smolen GA (1998). Suppressors of superoxide dismutase (SOD1) deficiency in Saccharomyces cerevisiae. Identification of proteins predicted to mediate iron-sulfur cluster assembly. J Biol Chem.

[163] Boillée S, Yamanaka K, Lobsiger CS, Copeland NG, Jenkins NA (2006). Onset and progression in inherited ALS determined by motor neurons and microglia. Science.

[164] Díaz-Amarilla P, Olivera-Bravo S, Trias E, Cragnolini A, Martínez-Palma L (2011). Phenotypically aberrant astrocytes that promote motoneuron damage in a model of inherited amyotrophic lateral sclerosis. Proc Natl Acad Sci U S A.

[165] Wang L, Gutmann DH, Roos RP (2011). Astrocyte loss of mutant SOD1 delays ALS disease onset and progression in G85R transgenic mice. Hum Mol Genet.

[166] Yamanaka K, Chun SJ, Boillee S, Fujimori-Tonou N, Yamashita H (2008). Astrocytes as determinants of disease progression in inherited amyotrophic lateral sclerosis. Nat Neurosci.

[167] Choi JS, Cho S, Park SG, Park BC, Lee DH (2004). Co-chaperone CHIP associates with mutant Cu/Zn-superoxide dismutase proteins linked to familial amyotrophic lateral sclerosis and promotes their degradation by proteasomes. Biochem Biophys Res Commun.

[168] Urushitani M, Kurisu J, Tateno M, Hatakeyama S, Nakayama K (2004). CHIP promotes proteasomal degradation of familial ALS-linked mutant SOD1 by ubiquitinating Hsp/Hsc70. J Neurochem.

[169] Ishigaki S, Niwa J, Yamada S, Takahashi M, Ito T (2007). Dorfin-CHIP chimeric proteins potently ubiquitylate and degrade familial ALS-related mutant SOD1 proteins and reduce their cellular toxicity. Neurobiol Dis.

[170] Watanabe M, Dykes-Hoberg M, Culotta VC, Price DL, Wong PC (2001). Histological evidence of protein aggregation in mutant SOD1 transgenic mice and in amyotrophic lateral sclerosis neural tissues. Neurobiol Dis.

[171] Zetterström P, Graffmo KS, Andersen PM, Brännström T, Marklund SL (2011). Proteins that bind to misfolded mutant superoxide dismutase-1 in spinal cords from transgenic amyotrophic lateral sclerosis (ALS) model mice. J Biol Chem.

[172] Durham HD, Roy J, Dong L, Figlewicz DA (1997). Aggregation of mutant Cu/Zn superoxide dismutase proteins in a culture model of ALS. J Neuropathol Exp Neurol.

[173] Roy J, Minotti S, Dong L, Figlewicz DA, Durham HD (1998). Glutamate potentiates the toxicity of mutant Cu/Zn-superoxide dismutase in motor neurons by postsynaptic calcium-dependent mechanisms. J Neurosci.

[174] Koyama S, Arawaka S, Chang-Hong R, Wada M, Kawanami T (2006). Alteration of familial ALS-linked mutant SOD1 solubility with disease progression: its modulation by the proteasome and Hsp70. Biochem Biophys Res Commun.

[175] Batulan Z, Taylor DM, Aarons RJ, Minotti S, Doroudchi MM (2006). Induction of multiple heat shock proteins and neuroprotection in a primary culture model of familial amyotrophic lateral sclerosis. Neurobiol Dis.

[176] Liu J, Shinobu LA, Ward CM, Young D, Cleveland DW (2005). Elevation of the Hsp70 chaperone does not effect toxicity in mouse models of familial amyotrophic lateral sclerosis. J Neurochem.

[177] Kalmar B, Novoselov S, Gray A, Cheetham ME, Margulis B (2008). Late stage treatment with arimoclomol delays disease progression and prevents protein aggregation in the SOD1 mouse model of ALS. J Neurochem.

[178] Kieran D, Kalmar B, Dick JR, Riddoch-Contreras J, Burnstock G (2004). Treatment with arimoclomol, a coinducer of heat shock proteins, delays disease progression in ALS mice. Nat Med.

[179] Arai T, Hasegawa M, Akiyama H, Ikeda K, Nonaka T (2006). TDP-43 is a component of ubiquitin-positive tau-negative inclusions in frontotemporal lobar degeneration and amyotrophic lateral sclerosis. Biochem Biophys Res Commun.

[180] Neumann M, Sampathu DM, Kwong LK, Truax AC, Micsenyi MC (2006). Ubiquitinated TDP-43 in frontotemporal lobar degeneration and amyotrophic lateral sclerosis. Science.

[181] Gitcho MA, Baloh RH, Chakraverty S, Mayo K, Norton JB (2008). TDP-43 A315T mutation in familial motor neuron disease. Ann Neurol.

[182] Kabashi E, Valdmanis PN, Dion P, Spiegelman D, McConkey BJ (2008). TARDBP mutations in individuals with sporadic and familial amyotrophic lateral sclerosis. Nat Genet.

[183] Sreedharan J, Blair IP, Tripathi VB, Hu X, Vance C (2008). TDP-43 mutations in familial and sporadic amyotrophic lateral sclerosis. Science.

[184] Kwiatkowski TJ, Bosco DA, Leclerc AL, Tamrazian E, Vanderburg CR (2009). Mutations in the FUS/TLS gene on chromosome 16 cause familial amyotrophic lateral sclerosis. Science.

[185] Vance C, Rogelj B, Hortobágyi T, De Vos KJ, Nishimura AL (2009). Mutations in FUS, an RNA processing protein, cause familial amyotrophic lateral sclerosis type 6. Science.

[186] Polymenidou M, Lagier-Tourenne C, Hutt KR, Huelga SC, Moran J (2011). Long pre-mRNA depletion and RNA missplicing contribute to neuronal vulnerability from loss of TDP-43. Nat Neurosci.

[187] Sephton CF, Cenik C, Kucukural A, Dammer EB, Cenik B (2011). Identification of neuronal RNA targets of TDP-43-containing ribonucleoprotein complexes. J Biol Chem.

[188] Tollervey JR, Curk T, Rogelj B, Briese M, Cereda M (2011). Characterizing the RNA targets and position-dependent splicing regulation by TDP-43. Nat Neurosci.

[189] Ling SC, Albuquerque CP, Han JS, Lagier-Tourenne C, Tokunaga S (2010). ALS-associated mutations in TDP-43 increase its stability and promote TDP-43 complexes with FUS/TLS. Proc Natl Acad Sci U S A.

[190] Kryndushkin D, Wickner RB, Shewmaker F (2011). FUS/TLS forms cytoplasmic aggregates, inhibits cell growth and interacts with TDP-43 in a yeast model of amyotrophic lateral sclerosis. Protein Cell.

[191] Wang JW, Brent JR, Tomlinson A, Shneider NA, McCabe BD (2011). The ALS-associated proteins FUS and TDP-43 function together to affect Drosophila locomotion and life span. J Clin Invest.

[192] Kim SH, Shanware NP, Bowler MJ, Tibbetts RS (2010). Amyotrophic lateral sclerosis-associated proteins TDP-43 and FUS/TLS function in a common biochemical complex to co-regulate HDAC6 mRNA. J Biol Chem.

[193] Gregory JM, Barros TP, Meehan S, Dobson CM, Luheshi LM (2012). The aggregation and neurotoxicity of TDP-43 and its ALS-associated 25 kDa fragment are differentially affected by molecular chaperones in Drosophila. PLoS One.

[194] Estes PS, Boehringer A, Zwick R, Tang JE, Grigsby B (2011). Wild-type and A315T mutant TDP-43 exert differential neurotoxicity in a Drosophila model of ALS. Hum Mol Genet.

[195] Orr HT (2012). Cell biology of spinocerebellar ataxia. J Cell Biol.

[196] Cummings CJ, Mancini MA, Antalffy B, DeFranco DB, Orr HT (1998). Chaperone suppression of aggregation and altered subcellular proteasome localization imply protein misfolding in SCA1. Nat Genet.

[197] Cummings CJ, Sun Y, Opal P, Antalffy B, Mestril R (2001). Over-expression of inducible HSP70 chaperone suppresses neuropathology and improves motor function in SCA1 mice. Hum Mol Genet.

[198] Warrick JM, Chan HY, Gray-Board GL, Chai Y, Paulson HL (1999). Suppression of polyglutamine-mediated neurodegeneration in Drosophila by the molecular chaperone HSP70. Nat Genet.

[199] Al-Ramahi I, Lam YC, Chen HK, de Gouyon B, Zhang M (2006). CHIP protects from the neurotoxicity of expanded and wild-type ataxin-1 and promotes their ubiquitination and degradation. J Biol Chem.

[200] Choi JY, Ryu JH, Kim HS, Park SG, Bae KH (2007). Co-chaperone CHIP promotes aggregation of ataxin-1. Mol Cell Neurosci.

[201] Jana NR, Dikshit P, Goswami A, Kotliarova S, Murata S (2005). Co-chaperone CHIP associates with expanded polyglutamine protein and promotes their degradation by proteasomes. J Biol Chem.

[202] Williams AJ, Knutson TM, Colomer Gould VF, Paulson HL (2009). In vivo suppression of polyglutamine neurotoxicity by C-terminus of Hsp70-interacting protein (CHIP) supports an aggregation model of pathogenesis. Neurobiol Dis.

[203] Tsai HF, Lin SJ, Li C, Hsieh M (2005). Decreased expression of Hsp27 and Hsp70 in transformed lymphoblastoid cells from patients with spinocerebellar ataxia type 7. Biochem Biophys Res Commun.

[204] Li L, Saegusa H, Tanabe T (2009). Deficit of heat shock transcription factor 1-heat shock 70 kDa protein 1A axis determines the cell death vulnerability in a model of spinocerebellar ataxia type 6. Genes Cells.

[205] Huen NY, Chan HY (2005). Dynamic regulation of molecular chaperone gene expression in polyglutamine disease. Biochem Biophys Res Commun.

[206] Bailey CK, Andriola IF, Kampinga HH, Merry DE (2002). Molecular chaperones enhance the degradation of expanded polyglutamine repeat androgen receptor in a cellular model of spinal and bulbar muscular atrophy. Hum Mol Genet.

[207] Waza M, Adachi H, Katsuno M, Minamiyama M, Sang C (2005). 17-AAG, an Hsp90 inhibitor, ameliorates polyglutamine-mediated motor neuron degeneration. Nat Med.

[208] Tokui K, Adachi H, Waza M, Katsuno M, Minamiyama M (2009). 17-DMAG ameliorates polyglutamine-mediated motor neuron degeneration through well-preserved proteasome function in an SBMA model mouse. Hum Mol Genet.

[209] Thomas M, Harrell JM, Morishima Y, Peng HM, Pratt WB (2006). Pharmacologic and genetic inhibition of hsp90-dependent trafficking reduces aggregation and promotes degradation of the expanded glutamine androgen receptor without stress protein induction. Hum Mol Genet.

[210] Adachi H, Katsuno M, Minamiyama M, Sang C, Pagoulatos G (2003). Heat shock protein 70 chaperone overexpression ameliorates phenotypes of the spinal and bulbar muscular atrophy transgenic mouse model by reducing nuclear-localized mutant androgen receptor protein. J Neurosci.

[211] Muchowski PJ, Schaffar G, Sittler A, Wanker EE, Hayer-Hartl MK (2000). Hsp70 and hsp40 chaperones can inhibit self-assembly of polyglutamine proteins into amyloid-like fibrils. Proc Natl Acad Sci U S A.

[212] Hay DG, Sathasivam K, Tobaben S, Stahl B, Marber M (2004). Progressive decrease in chaperone protein levels in a mouse model of Huntington’s disease and induction of stress proteins as a therapeutic approach. Hum Mol Genet.

[213] Wang AM, Morishima Y, Clapp KM, Peng HM, Pratt WB (2010). Inhibition of hsp70 by methylene blue affects signaling protein function and ubiquitination and modulates polyglutamine protein degradation. J Biol Chem.

[214] Baldo B, Weiss A, Parker CN, Bibel M, Paganetti P (2012). A screen for enhancers of clearance identifies huntingtin as a heat shock protein 90 (Hsp90) client protein. J Biol Chem.

[215] Adachi H, Waza M, Tokui K, Katsuno M, Minamiyama M (2007). CHIP overexpression reduces mutant androgen receptor protein and ameliorates phenotypes of the spinal and bulbar muscular atrophy transgenic mouse model. J Neurosci.

[216] Morishima Y, Wang AM, Yu Z, Pratt WB, Osawa Y (2008). CHIP deletion reveals functional redundancy of E3 ligases in promoting degradation of both signaling proteins and expanded glutamine proteins. Hum Mol Genet.

[217] Jana NR, Tanaka M, Wang Gh, Nukina N (2000). Polyglutamine length-dependent interaction of Hsp40 and Hsp70 family chaperones with truncated N-terminal huntingtin: their role in suppression of aggregation and cellular toxicity. Hum Mol Genet.

[218] Walter GM, Smith MC, Wisén S, Basrur V, Elenitoba-Johnson KS (2011). Ordered assembly of heat shock proteins, Hsp26, Hsp70, Hsp90, and Hsp104, on expanded polyglutamine fragments revealed by chemical probes. J Biol Chem.

[219] Jana NR, Nukina N (2005). BAG-1 associates with the polyglutamine-expanded huntingtin aggregates. Neurosci Lett.

[220] Danzer KM, Ruf WP, Putcha P, Joyner D, Hashimoto T (2011). Heat-shock protein 70 modulates toxic extracellular α-synuclein oligomers and rescues trans-synaptic toxicity. FASEB J.

[221] Riedel M, Goldbaum O, Schwarz L, Schmitt S, Richter-Landsberg C (2010). 17-AAG induces cytoplasmic alpha-synuclein aggregate clearance by induction of autophagy. PLoS One.

[222] Zhang YJ, Gendron TF, Xu YF, Ko LW, Yen SH (2010). Phosphorylation regulates proteasomal-mediated degradation and solubility of TAR DNA binding protein-43 C-terminal fragments. Mol Neurodegener.

[223] Jinwal UK, Abisambra JF, Zhang J, Dharia S, O’Leary JC (2012). Cdc37/Hsp90 protein complex disruption triggers an autophagic clearance cascade for TDP-43 protein. J Biol Chem.

[224] Katsuno M, Sang C, Adachi H, Minamiyama M, Waza M (2005). Pharmacological induction of heat-shock proteins alleviates polyglutamine-mediated motor neuron disease. Proc Natl Acad Sci U S A.

